# Dietetics1Read before the American Veterinary Medical Association, New York, September, 1899.

**Published:** 1899-11

**Authors:** W. H. Dalrymple

**Affiliations:** Baton Rouge, La.


### DIETETICS.[1]

By W. H. Dalrymple, M.R.C.V.S.,

BATON ROUGE, LA.

(Continued from page 634.)

When the digestible nutrients in a food-stuff are properly balanced, and a sufficient amount of food given for the purpose required of it, it is known as a "balanced ration;" or, in other words, a balanced ration may be said to be a combination of farm food products containing the various nutrients in such proportion and amount as will nourish the animal for a period of 24 hours, with the least waste of these nutritive elements. To know how to

[1] Read before the American Veterinary Medical Association, New York, September, 1899.

balance a ration is a very valuable piece of information for the veterinarian, in that on account of this knowledge the value to his client may be materially enhanced. I think it may be presumed that the majority of agriculturists, or stockowners in general, know very little of the actual feeding value of any of the food crops in common use. True, they may have access to tables of feeding standards showing the nutritive value of the various cereals, forage crops, etc., but is he, as a rule, competent to make the required calculation, even with such assistance? I think not, as a rule. He may have some idea as to what he is feeding for and the result he would wish to see accomplished, but how to obtain it in the most economic manner he knows not. He is as liable to feed a one-sided ration where it is contraindicated as he is to hit upon a correct one, which, of course, means loss—loss in the lack of development of his animal, and loss, also, in the waste of certain nutrients, which, had they been balanced with the required amount of others in which they were deficient, would have been a saving. It is a fact in my section of the country, and I presume there is not such a great deal of difference in others, that a want of more accurate knowledge on the subject of dietetics is responsible for more loss than any other connected with the business of the agriculturist. There is waste from giving more food than is necessary; there is loss of work from the lack of energy produced by giving too much or too little and at improper intervals. There is the dietetic disease brought about as sequelæ, and the fatalities which frequently result, and many other misfortunes to the stockowner through a want of a more accurate knowledge of the physiology of foods and how to intelligently utilize them.

Here, I think, the veterinarian could prove to his client that he is possessed of invaluable scientific knowledge along this line of great practical worth to the stockowner. Now, it is not suggesting to a farmer who may request information as to the balancing of a ration for his stock, to go and buy certain food-materials while he has others on his place of equal feeding value, that we are going to make our impression felt; but it is by instructing him how to utilize to the best advantage those he has by making a balanced ration out of them. This we ought all be able to do from the professional training in dietetics our modern schools afford. It has a telling effect when the veterinarian can impart information on a branch of a man's business of which he previously considered he knew all about, as a great many do, and it increases the appreciation of us, as a profession, by the people.

There is another point which, although of a more purely agricultural character, perhaps is worthy of being known by the veterinarian, more especially the country practitioner, and that is, the fertilizing value of feeding-stuffs. It is well known to the scientific agriculturist that the chief chemical constituents required in a fertilizer are nitrogen, phosphoric acid, and potash, because plants exhaust the soil of these more than of any of the others, which are usually in great abundance. Farm-yard manures, or domestic fertilizers, may benefit the soil because of the vegetable matter they contain acting as a mush and forming what is known as humus; but so far as being food for plants is concerned, their value rests upon their contents of the three constituents I have just mentioned.

Now, we learn from the investigations of Lawes and Gilbert, of Rothamstead, England, that the fattening steer retains only 3.9 per cent. of the nitrogen supplied in its food, voiding 22.6 per cent. in the solid excrement, and 73 per cent. in the urine; in all, 96.1 per cent. of the nitrogen supplied this animal in his food reappears in the excrement, and less than 4 per cent. is stored in the body. In this case it is, perhaps, due to the fact that during the fattening process the grown animal adds little to his body beside fat, which does not contain nitrogen. With the cow, 24.5 per cent. of the nitrogen of the feed is used mainly for the production of the casein and albumin of the milk, and 75 per cent. appears in the excrement. It is shown by the experiments on these and some other animals, that from 75 to 95 per cent. of all the fertilizing constituents of feeding-stuffs reappears in the solid and liquid excrement. When we take into consideration that the average commercial value of nitrogen is fifteen cents per pound, it is not an important piece of information to the farmer to know that certain food materials contain so much fertilizing value, and that by carefully saving the voidings he has from 75 to 95 per cent. left in it for use on the land after it has performed its function in the animal economy. This is, perhaps, a slight digression, but I merely alluded to it to emphasize the fact that we cannot be possessed of too much information that may be of practical value to us or to those with whom we come in contact while pursuing our professional calling.

I will just say a word or two on the subject of calorimetry connected with foods, or the measurement of the quantity of heat contained in them. This is an important study, as the theoretical heat-producing and mechanical value of food is in direct proportion to its feeding value. The term calorie is used to designate the amount of heat required to raise the temperature of one kilogramme of water 1° C., or one pound of water 4° F. If we use the foot-ton, which is the unit of mechanical energy, instead of the heat unit, the calorie then represents the energy required to raise a weight of 1.53 tons one foot high. For instance, in 1 gramme of protein there are 4.1 calories, or 6.3 foot-tons. In 1 gramme of ether extract, 9.3 calories, or 14.2 foot-tons; and in 1 gramme of carbohydrates, 4.1 calories, or 6.3 foot-tons. This means that when a gramme of protein, whether of body substance or furnished in feeding-stuffs, is consumed in the body, it will, if transformed into heat, yield enough heat to raise the temperature of 4.1 kilogrammes of water 1° C., or if transformed into mechanical energy, do the work executed by the steam-engine in raising a weight of one ton 6.3 feet, or 6.3 tons one foot, and so on. It is shown by the calories of food substances how much heat these will impart when utilized for that purpose by the animals, or the theoretical amount of work they can accomplish. The calories in feeding-stuffs reduced to available form may be stated thus, as given by Prof. Henry: In 1 gramme of digestible protein there are 4.1 calories; in 1 pound, 1860 calories. In a similar amount of digestible carbohydrates there are a similar number of calories, and in digestible fat, in 1 gramme there are 9.3 calories, and in 1 pound, 4220. By the use of these factors we can determine the potential energy of the digestible constituents of any feeding stuff.

It is not necessary, nor have we the time, to review in detail the best methods of feeding the different domestic animals; but I might mention that system is of the greatest importance. We are all familiar with the beneficial results of systematic feeding. It is difficult to lay down any hard-and-fast rule to be observed, as the practices in different parts of the country and of the world are so diverse and yet successful. I think, however, that the veterinarian should be guided by the anatomo-physiological requirements of the animal—a thing unknown to the greater part of the laity—the amount and character of the work, as well as the nutritive constituents of the food; but whatever the practice may be, so long as it is consistent with the requirements mentioned, uniformity and regularity should always prevail.

Not only is a one-sided food, so far as its nutritive elements are concerned, objectionable, but it is conceded, I think, by the best authorities, that a variety in food is more desirable than a short dietary, both for the human and the animal systems. By this we mean a mixing of a variety of food-stuffs, or substituting certain foods for others having the desired nutritive value to make the balance a correct one. It seems to be a universally recognized fact that oats are, *par excellence*, the best force-developing food for the horse, for instance; but there are a number of practical results recorded which go to show that a large percentage of a purely oat ration can be replaced with a mixture of other grains, and with great advantage, which has also been shown by experiments with other varieties of domesticated animals. In the case of the private individual, where only one or perhaps a pair of horses are kept, or in the racing stable, it may be more convenient to use the short bill of fare; but with the large business establishments requiring the work of a number of animals, or on the farm, the substitutional dietary may not only prove eminently satisfactory, so far as the health and working condition of the animals are concerned, but of great economic value also.

Most of you who are familiar with the more recent works on the subject of feeding must have observed the reference made to the rations fed by the various English and Continental European street-car and omnibus companies, in which is found a more or less mixed dietary. But perhaps the most convincing evidence of the advantage of a variety in feed is that given by Mr. Charles Hunting, M.R.C.V.S., of South Hetton, County Durham, England, in connection with 149 colliery horses. These animals, although receiving 168 pounds of whole oats and 154 pounds of hay each per week, were in a miserably poor condition, which necessitated a reduction in the amount of their work. This ration was changed to one containing 109 pounds of mixed crushed grains (peas, barley, oats, and bran) and 98 pounds of hay. Within three months from the date of the change the whole of these animals were in excellent health and condition, and were able to do more work than when first taken in hand. What were the results? There was, first of all, a saving in the amount of food-stuffs, the value of which, estimated per year, reached in the neighborhood of $18,000; there was a saving on the work of the digestive organs, between that required to digest 322 pounds and 207 pounds of feeding material, and there was a gain in the amount of work the animals were able to perform. This evidence may be taken as authentic. Mr. Hunting has always held a prominent position in the veterinary profession in England, and I believe he is looked upon as the pioneer of this system of feeding large studs of working horses in that country. Beside Mr. Hunting's results, however, I am personally familiar with others, more especially the London Tramway Company's horses, which, taken as a whole and when under the supervision of Prof. McGill, were considered the finest lot of animals engaged in similar work in Europe at least. In corrobration of this statement, I personally have witnessed a letter from Mr. Hunting to Prof. McGill to that effect.

Looking at the matter from an unprejudiced stand-point, and from the records of experiments and practical trials, I think the conclusion may be fairly drawn that if a properly balanced mixed diet produces as satisfactory results as the best single or monotonous one, and that such a considerable saving can be effected by its adoption, the system is certainly worthy of being brought into more general practice.

I have not touched upon the subject of water, which, of course, plays a very important part in successful dieting. I think, however, I may forego any extensive allusion to this aliment, except to remark that it should be palatable, pure, and wholesome; that it should be regulated as to quantity, according to the condition of the animal, and allowed at the proper time and intervals. In the case where animals have free access to water at all times, it will only be necessary to see that the first two conditions exist; they will regulate the time and quantity to suit themselves.

This subject is, as you are aware, an almost inexhaustible one, and it would be the grossest presumption to attempt to deal with it in other than in the most cursory manner in a paper of this kind. I have been forced to resort largely to generalities, which are always most unsatisfactory; but if I have, in generalizing, touched upon any points which may stimulate a revival of interest in this important topic in anyone present, I can assure you I will feel repaid for my effort.

---

The outcome of the Department of Agriculture in the work of conducting post-graduate studies for the development of scientific aids for the many departments is being watched with much interest. The fact of its being open only to those of advanced education through agricultural colleges and experiment station work, under the merit system of selection, makes the movement of greater interest and import, and the fact that the government scientific laboratories have long been the training-school for men for higher places and better salaries than the government offers will be perhaps a solution in a greater or lesser measure of always having trained men to fill these places when vacancies occur. In that it affords a certain compensation it will attract many young men of considerable talent, who, eager for a broader and better education, yet cannot always command the means to obtain it.

